# A Cooking School

**Published:** 1876-12

**Authors:** 


### A COOKING SCHOOL.

Miss Julia Corson, a well-known writer on domestic topics, has a school in full operation at No. 8 St. Marks Place, for ladies who desire to see practically illustrated by an expert, the preparation of various foods in an economical manner. Miss Corson has secured the services of an educated Italian *Chef*, M. Guiseppe Rudmanii, who performs all the operations before the class and gives exact recipes for all dishes, with the cost. These recipes are copied by the ladies, and by these aids, complete bills of fare, for two persons or a hundred, can readily be made up. Much valuable knowledge is imparted by the *Chef* in his careful and detailed explanation of each process. Happily, he excells as an instructor, not only on account of his excellence as an artist, but because of his ability to impart his knowledge to others. Added interest is given by the intelligent running commentary upon the various manipulations, by Miss Corson. Ladies who wish to know how to regulate their own tables, or who desire to have their daughters instructed in the preparation of all kinds of food, should avail themselves of this excellent opportunity. A few practical lessons here would be of more value than forty "cook-books."

